# Augmented Reality in Health Education: Transforming Nursing, Healthcare, and Medical Education and Training

**DOI:** 10.3390/nursrep15080289

**Published:** 2025-08-08

**Authors:** Georgios Lampropoulos, Pablo Fernández-Arias, Antonio del Bosque, Diego Vergara

**Affiliations:** 1 Department of Applied Informatics, School of Information Sciences, University of Macedonia, 54636 Thessaloniki, Greece; glampropoulos@uom.edu.gr; 2 Department of Education, School of Education, University of Nicosia, Nicosia 2417, Cyprus; 3 Technology, Instruction and Design in Engineering and Education Research Group (TiDEE.rg), Catholic University of Ávila, C/Canteros s/n, 05005 Ávila, Spain; pablo.fernandezarias@ucavila.es (P.F.-A.); antonio.bosque@ucavila.es (A.d.B.)

**Keywords:** augmented reality, AR, health education, health sciences, nursing education, healthcare education, medical education, scoping review, bibliometric analysis

## Abstract

**Background**: In health sciences education and particularly, in healthcare education, nursing education, and medical education, augmented reality is being increasingly used due to the changes and benefits to teaching and learning approaches it can yield. However, as the field advances, it is important to systematically map the current literature and provide an overview of the field. **Aim**: By analyzing the current literature, this study focuses on examining the use of augmented reality in healthcare, nursing, and medical education and training. **Method**: The study adopts a systematic mapping review approach and analyzes 156 studies that were published during 2010–2025. **Results**: The results revealed that augmented reality is an effective educational tool that can support teaching and learning of diverse subjects in the context of health education, as it enables learners to combine their theoretical knowledge with practical applications within interactive and immersive learning environments and simulations without risking patient safety. Increased learning outcomes, including hands-on acquisition of practical skills and clinical competencies, engagement, performance, knowledge gain and retention, as well as their critical thinking and decision-making were observed. The potential of augmented reality to offer realistic and interactive visual representations, to support procedural training, to provide cost-effective solutions, to enhance collaborative learning, and to increase accessibility to education, even in resource-limited settings, was highlighted. Education stakeholders expressed positive attitudes and perspectives toward the adoption and integration of augmented reality into health sciences education. **Discussion**: The results emphasize the role of augmented reality in supporting and improving health education. Additionally, the study revealed six main topics, identified current research gaps, and provided future research directions. **Conclusions**: When appropriately applied, augmented reality has the potential to effectively support and enrich nursing, healthcare, and medical education and training.

## 1. Introduction

Given the nature of health sciences education, it is not always feasible or applicable for learners to practice the complex skills required within real environments and settings due to safety, didactic, and cost reasons [1]. To overcome these barriers, immersive technologies, including augmented reality and virtual reality, are increasingly being used in healthcare, nursing, and medical education to engage students within immersive learning environments [2,3]. Specifically, through the use of computational units, augmented reality enhances the physical environment by overlaying digital content that users can perceive and interact with through various sensory channels [4,5,6,7].

Within augmented reality, immersive simulations and environments, which combine elements from both the physical and the virtual worlds, are being used to enrich the existing curriculum within health sciences education, to facilitate teaching and learning, and to enable learners to practice their social and practical skills [8,9,10]. The design and development of these immersive environments and simulations follow student-centered approaches [11] and within them, students can learn in a personalized manner [12]. Additionally, the use of augmented reality can result in the creation of new and effective content delivery methods and teaching approaches [13]. For example, the interactive and realistic virtual representations offered by augmented reality simplify and enhance the learning of complex concepts [9].

Since immersive technologies can offer secure virtual environments for learners to train that are more cost-effective and, in some cases, easier to create, these technologies are also increasingly being used in Low- and Middle-Income Countries (LMICs) [14]. However, suitable hardware devices, including head-mounted displays, should be utilized for their effective use [15]. Therefore, when it comes to their wider adoption, there are existing issues that should be resolved, including accessibility, the availability of educational resources, costs, and hardware limitations [3,14].

Studies that have focused on integrating augmented reality into health sciences have highlighted the educational benefits it can yield and its ability to effectively support different health sciences subjects when used as a teaching and learning tool [16,17,18,19]. Specifically, augmented reality provides immersive, situated, and ubiquitous learning environments for students to practice their practical skills in spatial context [20]. Hence, augmented reality enables students to acquire a more meaningful and direct experiential understanding of the concepts taught, as well as of the transferable and practical skills required [21]. As a result, students can develop different types of competencies by practicing their skills and improving their knowledge [2,19] within immersive environments, which leads to them achieving increased learning outcomes, engagement, content mastery, and satisfaction [9,22].

It is important to note that integrating augmented reality within health sciences education to enhance medical training and to increase learners’ knowledge and skills also leads to improvements in patients’ care [23]. Specifically, augmented reality has shown great potential “in clinical care delivery in patient care settings, in operating rooms, and inpatient settings, and in education and training of emergency care providers” and can also aid in telehealth through over distance care delivery [24]. Thus, augmented reality has the potential to transform and enrich the healthcare domain [11], supplement existing teaching and learning methods, and contribute to the development of healthcare professionals [25].

Furthermore, education stakeholders express positive views and attitudes regarding the use of augmented reality in health sciences education [9,18,19]. In addition, students reported a strong intention to use augmented reality in their learning, as they already have experience in using this technology in their daily life [26]. However, for their successful use within curricula, teachers should also embrace their adoption and be fully engaged in and committed to their integration [11,27].

The educational value of augmented reality can be further understood through the lens of established learning theories, which provide conceptual foundations for designing and evaluating augmented reality learning experiences. For example, constructivist theory emphasizes active, learner-centered knowledge construction, which aligns with the interactive and exploratory nature of augmented reality [28]. Similarly, situated learning theory promotes the use of authentic, context-rich, and interactive learning environments which augmented reality can offer through its immersive experiences and simulations [29,30]. Experiential learning theory further highlights the potential of augmented reality, as it enables learners to engage in hands-on, experiential learning activities that simulate real-world use cases within safe and controlled environments [31,32]. Additionally, Self-Determination Theory (SDT) emphasizes how learners’ motivation can be supported through the satisfaction of their basic psychological needs for autonomy, competence, and relatedness, something that augmented reality can provide through its interactive and personalized experiences that promote students’ sense of agency and engagement [33].

Immersive technologies have shown great potential to support health education [34]. Although the outcomes of recent studies have shown increased or positive effects in terms of performance and learning outcomes when using immersive technologies [35], there is a need for standardized assessment methods to be developed to identify key aspects for their successful integration in education curricula and determine best practices [36,37,38]. Additionally, there is a need to develop suitable pedagogical strategies and theoretical frameworks to effectively design, develop, and use augmented reality applications in health sciences education [19,39].

Despite the fact that the findings of recent review studies [2,14,15,18,19,24,25,35,38] showcase the transformative potential of augmented reality in improving educational outcomes within health sciences education, there is no study that systematically maps the field. Given the rapid advancement of the field, the necessity for such a study increases. Additionally, due to the multidisciplinary nature of the field, most studies focus on specific areas or approaches. Hence, there is a need for a study that can more broadly explore these concepts. Therefore, to address these literature gaps, this study examines the adoption and use of augmented reality in health education, focusing on healthcare, nursing, and medical education. Specifically, it follows a systematic mapping review approach and focuses on a bibliometric analysis and scientific mapping of the related field. In Section 2, we present the methodology used. In Section 3, we explore the results analysis and further discuss and analyzed these results in Section 4. Finally, the conclusions drawn, the implications, the research gaps, and suggestions for future research are provided in Section 5.

## 2. Materials and Methods

To ensure transparency and reproducibility, this study followed the “Preferred Reporting Items for Systematic Reviews and Meta-Analyses” (PRISMA 2020) guidelines [40]. This approach is suitable for examining both strictly defined and broadly defined topics [41,42], as is the case for this study, which explores the utilization of augmented reality in health education. Scopus and Web of Science were chosen to identify related documents due to their scientific rigor and their common use in similar studies [43,44]. Additionally, due to its performance and its prevalence in similar studies, Bibliometrix was selected to examine the document collection [45].

Since this study aimed to provide a broader perspective about the adoption and integration of augmented reality in nursing education, healthcare education, and medical education, more generic terms were used within the search query. Through this approach, it is better ensured that the initial identification of documents will not be limited or headed for a specific direction through the use of keywords. The following query was executed in both databases to search through both the document title and abstract fields: (“augmented reality” OR “AR”) AND (“healthcare” OR “health care” OR “medical” OR “medicine” OR “nursing”) AND (“education”)”. The search was restricted to relevant documents published after 2009.

**Figure 1:**
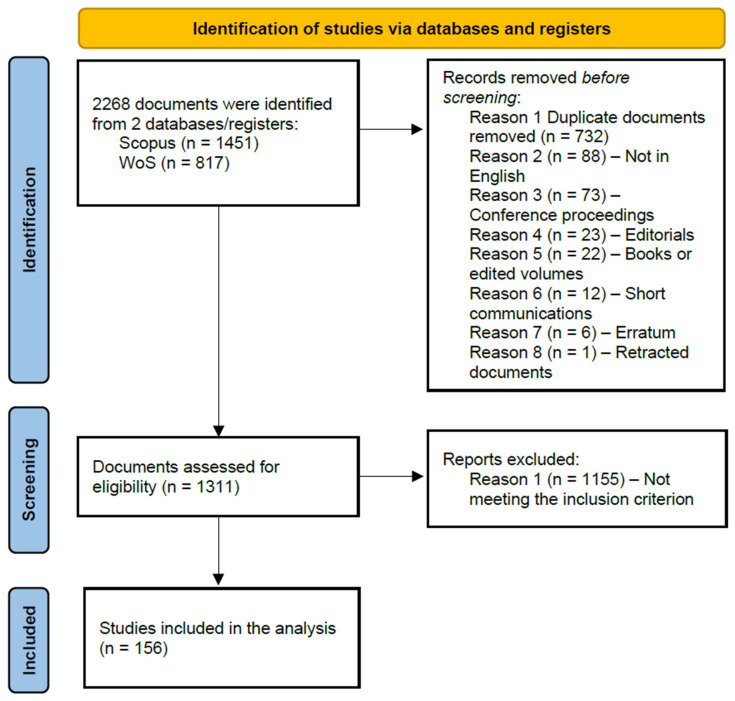
PRISMA flowchart.

Based on the PRISMA flowchart (Figure 1), 2268 documents were retrieved, with 1451 documents deriving from Scopus and 817 documents deriving from Web of Science. In total, 732 duplicate documents were identified and removed, and as a result, 1536 documents were further examined. Of these, 225 documents were excluded based on reasons 2–7 listed in Figure 1. The remaining 1311 documents were examined for inclusion. Given the scope of this study, the inclusion criteria required documents to primarily focus on the adoption and integration of augmented reality in nursing, medical, and/or healthcare education. In total, 1155 documents were removed for failing to meet the inclusion criteria. These documents were out of scope, since they did not involve augmented reality and/or did not focus on or were not applied in educational settings, and/or did not involve healthcare, nursing, or medical education. As a result, this study analyzed a final set of 156 documents.

## 3. Analysis of the Results

To examine the specifications and the characteristics of the document collection created, Bibliometrix was utilized. Table 1 presents the main attributes of the 156 documents explored. Two different time periods were used to calculate the annual growth rate. The first time period focused on documents published between January 2010 and December 2024, showing an annual growth rate of 29.17%, while the second time period included data from January 2010 to May 2025 and had an annual growth rate of 14.87%. The steady increase in annual growth rate, combined with a low average document age of 3.85 years, points to its growing relevancy in the field of health education.

Additionally, the documents were published by 681 authors in 123 sources. Each document was written by an average of 4.72 authors and received 28.83 citations on average. Among the 156 documents, only 3 were single-authored documents (1.9%). The documents mostly reflected articles that were published in journals (n = 67, 42.9%), followed by those that were published in conference proceedings (n = 46, 29.5%). Several studies were also classified as review studies (n = 38, 24.4%), while only a few documents were classified as being chapters within books or edited volumes (n = 5, 3.2%). Finally, a total of 41 countries were represented among the contributing authors, and the rate of international co-authorship was 16.03%.

**Figure 2:**
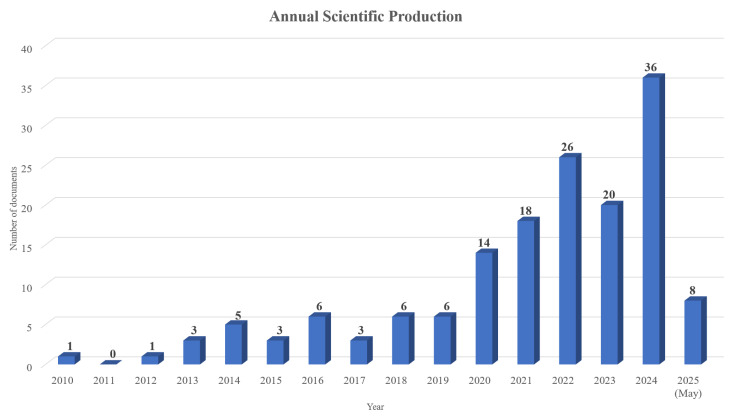
Number of documents published per year.

An analysis of annual scientific production (Figure 2) reveals an increase in the number of publications starting from 2020, with the most documents being published in 2024 (n = 36) and in 2022 (n = 26). However, there was a slight decline in publications in 2023. Hence, although there was a significant increase in the number of documents published in 2024, the current rate of published documents suggests that the topic is still in its infancy and further examination of the advances within the fields of healthcare, nursing, and medical education is required to explore the recent advances in augmented reality. Additionally, according to the results depicted in Table 2, the three documents published in 2017 have been highly impactful in shaping up this domain both in terms of the “mean total citations per document (MeanTCperDoc)” and the “mean total citations per year (MeanTCperYear)”. When considering the related outcomes, the “number of documents published each year (N)”, and the “citable years of the documents (CitableYears)”, it can be inferred that the documents published in 2021, 2019, and 2016 have also had a great impact. However, as the field matures and grows, these outcomes are expected to change.

Furthermore, Bradford’s law was applied to identify the most prominent sources based on the number of published documents. All types of sources (journals, conferences/proceedings, and edited book series) were assessed. In total, three clusters of sources were created. In particular, Cluster 1 consisted of 20 sources (16.3%), Cluster 2 consisted of 52 sources (42.3%), and Cluster 3 consisted of 51 sources (51.5%). These clusters take the number of documents published into account; therefore, there is an almost equal distribution of published documents among the three clusters, with Cluster 1 having 53 documents (34.0%), Cluster 2 having 52 documents (33.3%), and Cluster 3 having 51 documents (32.7%). To identify the most relevant sources, solely based on the number of documents published, the sources of Cluster 1 were further explored. The results are showcased in Table 3, where the “name of the sources (Source)”, the “ranking of the sources (Rank)”, the “number of published documents (Freq)”, the “cumulative number of published documents (cumFreq)”, and the “cluster (Cluster)” are presented. “*Cureus Journal of Medical Science*”, “*Anatomical Sciences Education*”, “*Frontiers in Virtual Reality*”, “*IEEE Conference on Virtual Reality and 3D User Interfaces Abstracts and Workshops (VRW)*”, “*Journal of Medical Internet Research*”, and “*Nurse Education Today*” were the sources that published three or more related documents. Additionally, there were 14 sources that published two documents each. Given the limited number of sources that have published on this topic more extensively, there is little change when examining the sources that have the highest h-index. Since the vast majority of sources had an h-index of less than two and 12 sources had an h-index of two, Table 3 presents only the sources that have an h-index of three or higher.

Following Lotka’s law, the distribution of authors based on their contributions to the topic was analyzed. The majority of authors (n = 635, 63.2%) participated in only one relevant publication, whereas a small number of authors contributed to three or more studies. Specifically, three authors contributed to three studies, two authors to four studies, and only one author to five studies. Considering the total number of documents published and the average of 4.72 co-authors per document, there is a clear need to foster deeper collaborative efforts.

**Figure 3:**
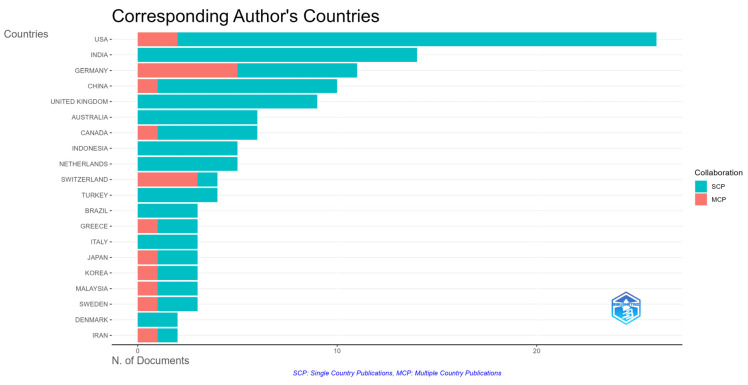
Publications and collaborations by country.

Taking into account the diversity of authors’ affiliations and countries and the international co-authorship rate, an analysis was conducted to identify the most active countries in this field. The country categorization and grouping provided by Bibliometrix were employed, using the corresponding author’s country or, if no corresponding author was listed, the country of the first author was used. The results, summarized in Table 4 and Figure 3, present the “countries (Country)”, the “number of documents published (Documents)”, and the extent of “intra-country (SCP)” and “inter-country (MCP)” collaborations, thus highlighting the leading countries in terms of publication output on this topic.

Specifically, the countries which contributed at least four documents are presented in Table 5. Among the countries, the United States, India, Germany, China, and the United Kingdom have published the most documents, followed by Australia, Canada, Indonesia, the Netherlands, Switzerland, and Turkey. Seven more countries contributed three documents each. Moreover, the United States and India had the highest SCP, while Germany had the highest MCP.

**Figure 4:**
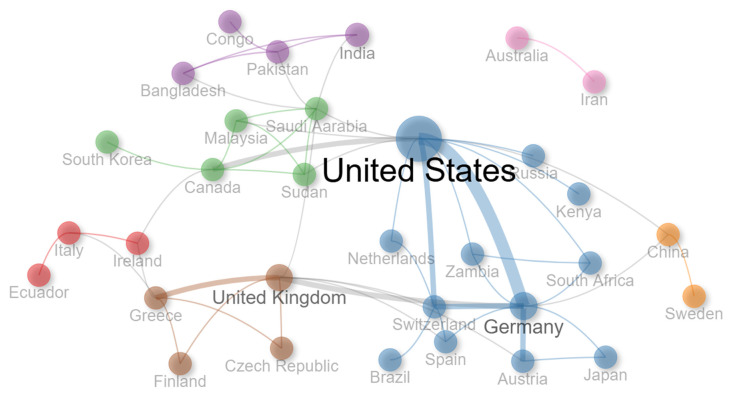
Country collaboration network.

When examining the total number of citations as well as the average document citations (Table 5), documents from Australia had the highest total citations and the highest average document citations, followed by the Netherlands. Once again, the fact that countries from different continents emerged as having published the most documents and are receiving the most citations further reinforces the global interest in the adoption and integration of augmented reality in health education. Nonetheless, as can be seen in Figure 4 and based on the international co-authorship rate (16.03%), it becomes evident that there is a need to strengthen international collaboration to further pursue and advance this multidisciplinary field.

## 4. Discussion

Augmented reality can yield several benefits in the educational domain; hence, its use in teaching and learning activities is rapidly advancing. Due to its ability to blend the virtual with the physical environment, augmented reality can create effective learning experiences that can be tailored to learners’ unique characteristics [46,47], increase learning outcomes and experiences [2,9,19,22], enhance social and practical skills [8,10], and offer adaptive and personalized learning experiences [10]. Augmented reality technology offers a unique approach to situated learning, particularly in health sciences education, where education is commonly rooted in real-life contexts, which can enhance learning experiences and effectively support a wide range of subjects, making it a valuable educational means for teaching and learning in this field [16,17,18,19]. Hence, several studies have examined its role in health sciences education [13,25] as well as its subfields, including medical education [1,2,9,12,14,15,16,20,23,24,35,38], nursing education [11,18,22,26], and healthcare education [3,8,17,19,21,39].

Given the increasing interest in adopting and using augmented reality in health sciences education and its multidisciplinary nature, this study put emphasis on exploring the existing literature through scientific mapping and bibliometric analysis. These approaches were selected as they can provide a sound representation of the current research landscape. Hence, the most highly cited documents (documents with over 100 citations) of the collection were also examined. The specific information is provided directly from the two databases. Table 6 presents the related studies.

Moro et al. [48] looked into the efficiency of using augmented and virtual reality in medical anatomy and health sciences education. The outcomes of their experiment involving 59 participants revealed that using augmented reality is equally as impactful and effective as using virtual reality and tablet devices; however, when using augmented reality, students did not exhibit adverse effects. Additionally, augmented reality was proven to be a technology that can supplement existing anatomical education and that can promote students’ intrinsic motivation. The systematic review of Barsom et al. [49] focused on the effectiveness of augmented reality interventions in the field of medical training. Through the analysis of 27 related studies, it was revealed that most augmented reality applications targeted laparoscopic surgical training, neurosurgical procedures training, and echocardiography training. Augmented reality applications showcased great potential in supporting medical training and can influence the healthcare domain; however, it was highlighted that more experimental studies must be conducted and that uniform and reliable assessment methods and strategies need to be developed.

Kamphuis et al. [1] explored how augmented reality can be utilized in medical education. In their study, they identified key applications of augmented reality, described augmented reality learning environments, and commented upon future research directions. Finally, they revealed the need to further examine the motivational value of augmented reality, as well as its influence on learners’ psychomotor skills and visualization skills. Barteit et al. [2] conducted a systematic literature review focusing on augmented reality, virtual reality, and mixed reality-based head-mounted devices in medical education. The results of the analysis of 27 students highlighted that most studies adopted augmented reality and virtual reality in anatomy and surgery education. Additionally, it was revealed that immersive technologies can effectively support medical education while increasing students’ motivation and engagement. Finally, students become more enthusiastic about and interested in the learning process when learning using head-mounted display interventions. In their systematic literature review, which analyzed 16 relevant studies, Joda et al. [50] looked into how virtual and augmented reality can be adopted and integrated into dental and medical education. These technologies had a positive influence on learning experiences and outcomes and were mostly used to support clinical testing, motor skills training, learning of concepts, and treatment. This study also highlighted the need for more experimental case studies to assess the long-term influence of these technologies and the need for the outcomes of related studies to be examined through a meta-analysis to understand their influence on dental medicine and health sciences education.

Following an integrative review approach, Zhu et al. [19] examined the role and use of augmented reality in healthcare education. The analysis of the 25 studies examined revealed the potential of augmented reality to effectively support various topics within healthcare education. Additionally, education stakeholders express positive attitudes toward its use and its potential. This study calls upon the need for future augmented reality applications that target the healthcare education domain to focus more on the integration of clinical competencies to ensure the safety of patients. Furthermore, in their integrative review, Gerup et al. [17] examined the influence of using augmented and mixed-reality applications into healthcare education. Their outcomes highlighted the potential of augmented reality to positively influence health sciences education. Most of the applications examined were established and focused on anesthesia and anatomy. Additionally, several educational benefits were observed when using augmented reality; for example, students who learnt through augmented reality applications significantly outperformed learners who learnt through traditional methods in terms of performance. Their study comments upon the need to focus on identifying suitable instructional objectives and research designs for augmented reality and mixed-reality applications. Moreover, Moro et al. [25] conducted a meta-analysis and systematic literature review of 58 studies examining the application of virtual reality and augmented reality in medical and health sciences education. The findings showed a slight improvement in students’ performance when learning with augmented reality. Although the performance gains were not statistically significant, the authors suggested that augmented reality has the potential to serve as a viable and effective alternative to traditional teaching methods.

In their systematic literature review of 128 studies, Tang et al. [51] examined the use of augmented reality and other immersive technologies in medical education and training. They highlighted that immersive technologies are more commonly adopted to aid students in learning anatomy or surgery-related concepts. Based on their outcomes, augmented reality constitutes an effective educational means that can support medical practices and education. Dhar et al. [9] focused on the integration of augmented reality into medical education and examined students’ learning outcomes and experiences. Their outcomes highlight the potential of augmented reality to influence students’ practical skills, social skills, knowledge acquisition, and comprehension of complex concepts. However, the authors also indicated key limitations that need to be resolved, including cost, lack of available resources, accessibility, equitability, and social isolation. Finally, focusing on the domain of medical and health sciences education, Moro et al. [13] examined the influence of augmented reality applications. When examining students’ scores, their randomized controlled trial of 38 participants revealed no significant differences. Students expressed feelings of dizziness when using a head-mounted device; however, no other adverse health effects were observed. Hence, in both cases, augmented reality interventions emerged as effective in terms of learning and their potential to enrich existing teaching methods arose.

**Figure 5:**
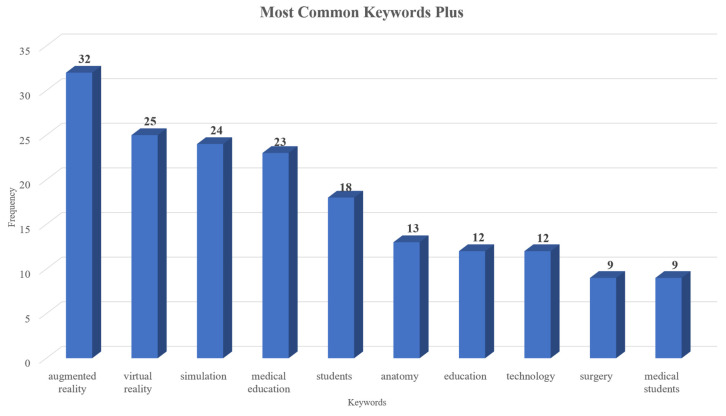
Most commonly used keywords according to Keywords plus.

**Figure 6:**
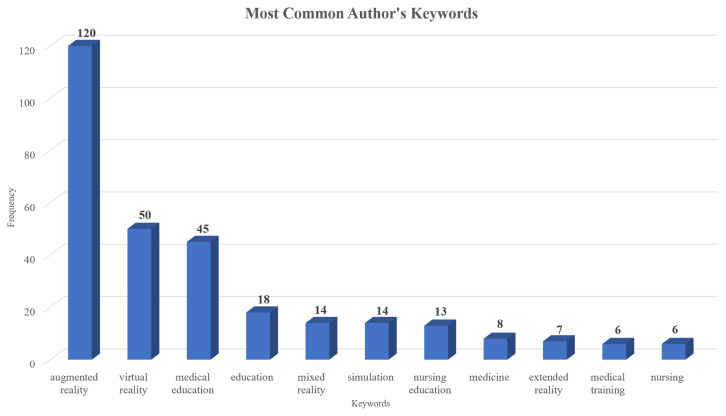
Keywords most commonly used by authors.

**Figure 7:**
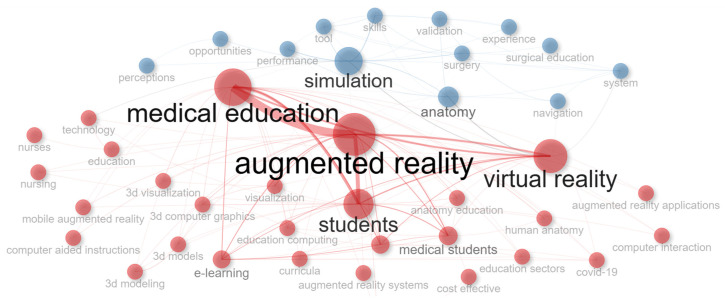
Co-occurrence network.

Furthermore, to identify the main topics discussed in the document collection, both “keywords plus” and “authors’ keywords” were analyzed. Figure 5 displays the most frequently occurring keywords plus which were “augmented reality”, “virtual reality”, “simulation”, “medical education”, “students”, “anatomy”, “education”, “technology”, “surgery”, and “medical students”. “Augmented reality”, “virtual reality”, “medical education”, “education”, “mixed reality”, “simulation”, “nursing education”, “medicine”, “extended reality”, “medical training”, and “nursing” were the keywords most frequently used by the authors (Figure 6). Furthermore, Figure 7 presents the co-occurrence network of the keywords in which two main clusters arose.

These outcomes further confirm and extend those of the existing literature, as the main emphasis is mostly placed on the domains of medical education, followed by nursing education, and to a lesser extent, on healthcare education. The keywords also highlight the close connection among extended reality technologies and show that these technologies are studied both independently and in combination. Additionally, studies mainly focus on supporting students’ anatomy and surgery education through augmented reality interventions. Emphasis is also put on immersive technologies, 3D environments, and immersive simulations to aid health sciences learners.

Focusing on the keywords, topics defined within the document collection were identified. As can be seen in Table 7, the keyword analysis revealed the following six main topics: (i) “Immersive technologies in health sciences education”, (ii) “Medical and anatomical education”, (iii) Simulation-based learning, (iv) “Nursing education”, (v) “Surgical and clinical training”, and (vi) “Immersive representations and experiences”. As can be seen from the topics, through the immersive and interactive learning experiences it can yield, augmented reality can influence various sectors of health sciences education. In addition to augmented reality, other immersive technologies, such as virtual reality, mixed reality, and the metaverse, can also influence teaching and learning in health sciences education [2,48,52,53]. Additionally, the immersive simulations offered by augmented reality allow learners to practice and improve their skills through hands-on activities [54]; hence, they are increasingly being used for clinical and surgical training. Learners can also acquire a better understanding of the complex concepts taught through the interactive, realistic, and immersive representations offered; thus, augmented reality is being used to support anatomical, nursing, and medical education. Immersive technologies, including both augmented reality and virtual reality, have shown great potential to be used in conjunction with exergames to further enhance the teaching and learning of health-related subjects, including rehabilitation, physical fitness, promotion of healthy habits, chronic disease management, and patient engagement in preventive healthcare [55].

The results of this study offer implications for educators, researchers, and policymakers in health education. Specifically, the integration of augmented reality into nursing, medical, and broader healthcare education offers a pedagogically sound way to bridge the gap between theoretical knowledge and practical application. By enabling learners to engage with realistic, immersive simulations in risk-free environments, augmented reality can enhance clinical preparedness, support the development of procedural and decision-making skills, and improve learners’ overall confidence. The study also highlights the potential of augmented reality to increase access to high-quality education by offering cost-effective and scalable solutions to address differences in clinical training opportunities, infrastructure, and faculty expertise. The positive attitudes of both students and educators toward augmented reality suggest a readiness and willingness to adopt innovative technologies, given that adequate support, training, and institutional frameworks are in place.

The six main topics identified highlight the broad and transformative potential of augmented reality across various dimensions of health education. These themes suggest that augmented reality can enhance both technical and non-technical skill development by providing interactive, visual, and experiential learning environments. Its ability to simulate complex procedures, visualize anatomical structures, and recreate real-world clinical contexts supports more effective teaching and learning, particularly in skills-based disciplines, such as nursing and surgery. Furthermore, the immersive nature of augmented reality enables empathetic and patient-centered training by exposing learners to simulated patient perspectives and conditions.

The implications of these findings point to the need for intentional integration of augmented reality into curricula, education training programs, and institutional planning to ensure its pedagogical value is fully realized across diverse health education settings. Additionally, the findings can offer insights for curriculum designers, educators, and institutional leaders seeking to modernize health education. Augmented reality has the potential to be effectively integrated into blended or hybrid instructional models, enriching traditional teaching with immersive, experiential components. For curriculum design, this means developing augmented reality-enhanced modules that align with learning outcomes in areas such as clinical reasoning, procedural training, and patient interaction. Development programs for educators should focus on building their digital pedagogical competencies, including the selection, implementation, and evaluation of augmented reality tools and applications. Institutions may also consider investing in suitable infrastructure and partnerships to create customized content tailored to their educational needs. As the field continues to evolve, such investments can position institutions at the forefront of innovative, accessible, and student-centered health education.

Finally, these findings highlight the value of developing standardized evaluation frameworks to more systematically assess the educational effectiveness, usability, and ethical dimensions of augmented reality-based learning in health education. While interest in the integration of augmented reality in health education continues to grow, current evaluation practices remain varied and are often limited by a lack of theoretical grounding, consistent methodologies, and long-term evidence. Clear and consistent evaluation standards could help ensure that augmented reality tools and applications are adopted based on their pedagogical value rather than their perceived innovation alone. Addressing these areas can support evidence-based adoption and guide future innovations in health professions education.

## 5. Conclusions

Following the PRISMA framework, this study examined the current literature, focusing on the utilization of augmented reality in health education. A document collection comprising 156 relevant studies was analyzed. The related documents were authored by authors from 41 countries and published across 123 different sources between 2010 and 2025. The document collection showcased a 29.17% annual growth rate of documents published, with most publications taking place in 2024. The main specifications of the documents, the sources, the number of annual publication and citations, as well as authors’ countries and affiliations were explored. The United States, India, Germany, China, and the United Kingdom produced the most publications, while studies from Australia, the Netherlands, Switzerland, and Denmark received the highest average number of citations per document. Emphasis was also placed on the document keywords and their analysis revealed six main topics: (i) “Immersive technologies in health sciences education”, (ii) “Medical and anatomical education”, (iii) Simulation-based learning, (iv) “Nursing education”, (v) “Surgical and clinical training”, and (vi) “Immersive representations and experiences”. Additionally, studies mostly focused on medical education, followed by nursing education, and, to a lesser extent, on healthcare education. Particular emphasis has been placed on the domains of anatomy education and surgery education, highlighting the need to further explore other related topics and subjects in depth.

According to the results, augmented reality arose as an educational tool which allows learners to combine their theoretical knowledge with practical applications. Augmented reality provides realistic and interactive visual representations, supports procedural training, offers cost-effective solutions, and increases accessibility to education even in resource-limited settings. Hence, it can support the teaching and learning of a wide range of health sciences subjects, including in LMICs. Its interactive and immersive simulations provide learners with opportunities to develop and practice clinical skills and patient interactions in safe environments that involve no risk for actual patients.

Additionally, the interactive and immersive environments and simulations provided by augmented reality allow learners to practice and develop their skills and patient interactions in a secure, safe, and controlled environment, without risking patient safety. Learning in such settings can increase students’ learning outcomes, engagement, performance, comprehension, hands-on acquisition of practical skills and clinical competencies, knowledge gain and retention, as well as their critical thinking and decision-making. Augmented reality also provides ubiquitous and personalized learning experiences and supports collaborative learning. Education stakeholders, including both teachers and students, express positive perspectives and attitudes regarding the use of augmented reality in health sciences education.

Besides the identified benefits, several challenges and limitations were also observed, which can be broadly categorized as pedagogical, technical, and institutional. Pedagogical challenges include difficulties in identifying appropriate instructional objectives, integrating augmented reality effectively into existing curricula, assessing the impact of augmented reality on student learning, aligning augmented reality content with learning outcomes, and ensuring that augmented reality-based activities promote deep and meaningful learning. Technical challenges relate to software and hardware limitations, including compatibility issues, connectivity issues, usability concerns, and the availability of reliable educational content. Institutional challenges encompass issues such as cost, accessibility, equitability, lack of infrastructure, and the risk of social isolation. Additionally, the need for structured training programs and ongoing professional development to prepare and support educators in implementing augmented reality in their classrooms was emphasized. Concerns regarding reduced face-to-face interaction and increased screen time were also noticed. Finally, the lack of institutional support or policy frameworks for adopting emerging technologies was highlighted. Beyond these challenges, the pedagogical and ethical implications of augmented reality require a closer examination. From a pedagogical perspective, there is a risk that the novelty of augmented reality could overshadow sound instructional design, leading to superficial engagement rather than meaningful learning gains. Educators may also lack theoretical frameworks or evidence-based models to guide the effective integration of augmented reality which, in turn, can result in its inconsistent or ineffective use. From an ethical perspective, concerns arise regarding data privacy and security, especially when augmented reality applications can collect user data or use biometric feedback. Issues related to the collection and processing of student data should be carefully addressed. On an institutional level, differences in access to augmented reality tools and application could increase existing educational inequalities. Without institutional policies that promote equitable access, suitable guidelines, professional development, and responsible use, augmented reality may reinforce rather than reduce disparities in education.

This study has some limitations that should be acknowledged. The bibliometric analysis was limited to publications indexed in only two databases which, while comprehensive, may not capture all relevant literature, particularly from regional or discipline-specific sources. Only English-language documents were included, potentially excluding valuable contributions published in other languages. Additionally, as this study employed a systematic mapping review. While this method allows for a broad overview of publication trends, thematic structures, and research patterns, it does not provide detailed insights into the quality or findings of individual studies. Nonetheless, the selected approach provides a useful overview of the development of the field and can aid future research.

On this note, future studies should concentrate on the development of uniform, robust, and reliable assessment methods and strategies, frameworks, and policies, as well as curriculum design and pedagogical approaches. Additional experimental studies need to be conducted to explore both the motivational benefits and long-term outcomes of augmented reality, providing more robust evidence to support its efficacy. Other important areas that need to be explored further include emergency nursing, interprofessional education, pediatrics, mental health training, geriatric care, anatomy and physiology instruction, and disaster preparedness. Future studies could also investigate how augmented reality can enhance clinical decision-making in high-pressure scenarios, facilitate collaboration among healthcare professionals, or improve patient communication skills in sensitive populations, such as children or older adults. Similarly, there is a need for more focus on specific health education studies that systematically and critically assess the outcomes and contents of the related studies (e.g., classifying augmented reality interventions; comparing the outcomes with the different professional profiles of the participants; categorizing research aims, methods, and findings; and creating a typology of the related studies). Having common standards and more experimental studies that focus on long-term effects would reduce the influence of the methodological diversity and sample size differences and allow for the development of relevant meta-analysis studies that further examine the impact of augmented reality on health sciences education. Future studies should also emphasize addressing privacy and ethical concerns and combining augmented reality with new technologies and approaches, including artificial intelligence, the Internet of Things, haptic devices, and gamification. Finally, how augmented reality can influence curriculum design and educational material development should be further examined.

Consequently, this study highlights the potential of augmented reality to enrich and transform existing healthcare education, nursing education, and medical education while also increasing learning outcomes. Through augmented reality, health sciences education and training can be enhanced which, in turn, will result in more qualified and better equipped health professionals and trainees and improved patient care and safety.

## Figures and Tables

**Table 1:** Document collection details.

Description	Results	Description	**Results**
**Main information about data**		**Document** types	
Timespan	2010:2025	Journal articles	67
Sources (journals, books, etc.)	123	Book chapter	5
Documents	156	Conference/Proceedings paper	46
Annual Growth Rate % (without including 2025)	29.17	Review	38
Annual Growth Rate % (including 2025)	14.87	Authors	
Document Average Age	3.85	**Authors**	681
Average Citations per Document	28.83	Authors of single-authored docs	3
References	3754	**Authors collaboration**	
**Document contents**		Single-authored docs	3
Keywords Plus (ID)	466	Co-Authors per Doc	4.72
Author’s Keywords (DE)	416	International co-authorships %	16.03

**Table 2:** Annual scientific production and citations.

Year	Mean TC per Doc	N	Mean TC per Year	Citable Years
2010	92	1	5.75	16
2011	-	-	-	-
2012	14	1	1	14
2013	59	3	4.54	13
2014	78.2	5	6.52	12
2015	34	3	3.09	11
2016	97.5	6	9.75	10
2017	172.67	3	19.19	9
2018	42.5	6	5.31	8
2019	60.67	6	8.67	7
2020	32	14	5.33	6
2021	40.5	18	8.1	5
2022	20.77	26	5.19	4
2023	7.65	20	2.55	3
2024	3.61	36	1.8	2
2025	0	8	0	1

**Table 3:** Key sources identified through Bradford’s law.

Source	Rank	Freq.	cumFreq.	Cluster
“*Cureus Journal of Medical Science*”	1	7	7	1
“*Anatomical Sciences Education*”	2	6	13	1
“*Frontiers in Virtual Reality*”	3	3	16	1
“*IEEE Conference on Virtual Reality and 3D User Interfaces Abstracts and Workshops (VRW)*”	4	3	19	1
“*Journal of Medical Internet Research*”	5	3	22	1
“*Nurse Education Today*”	6	3	25	1

**Table 4:** Country publication.

Country	Documents	SCP	MCP	Freq.	MCP_Ratio
United States	26	24	2	0.167	0.077
India	14	14	0	0.09	0
Germany	11	6	5	0.071	0.455
China	10	9	1	0.064	0.1
United Kingdom	9	9	0	0.058	0
Australia	6	6	0	0.038	0
Canada	6	5	1	0.038	0.167
Indonesia	5	5	0	0.032	0
Netherlands	5	5	0	0.032	0
Switzerland	4	1	3	0.026	0.75
Turkey	4	4	0	0.026	0

**Table 5:** Citation counts by country.

Country	Total Citations	Average Document Citations
Australia	865	144.2
Netherlands	607	121.4
United States	605	23.3
Germany	535	48.6
China	401	40.1
Switzerland	303	75.8
Sweden	187	62.3
United Kingdom	183	20.3
Denmark	144	72
Canada	77	12.8

**Table 6:** Most cited documents.

Document	DOI	Total Citations	Total Citations Per Year	Normalized Total Citations
[48]	10.1002/ase.1696	499	55.44	2.89
[49]	10.1007/s00464-016-4800-6	347	34.7	3.56
[1]	10.1007/s40037-013-0107-7	234	19.5	2.99
[2]	10.2196/29080	231	46.2	5.7
[50]	10.1016/j.compbiomed.2019.03.012	175	25	2.88
[19]	10.7717/peerj.469	143	11.92	1.83
[17]	10.5116/ijme.5e01.eb1a	138	23	4.31
[25]	10.1002/ase.2049	129	25.8	3.19
[51]	10.1016/j.edurev.2021.100429	117	29.25	5.63
[9]	10.1080/10872981.2021.1953953	112	22.4	2.77
[13]	10.1111/bjet.13049	106	21.2	2.62

**Table 7:** Key topics.

Topics	Related Keywords
Immersive technologies in health sciences education	augmented reality, virtual reality, mixed reality, extended reality, immersive technologies, technology, immersive technology, immersive learning.
Simulation-based learning	simulation, simulation training, surgical simulation, simulator, soft tissue simulation, simulation-based learning, interactive simulations.
Medical and anatomical education	medical education, anatomy, gross anatomy education, human anatomy, anatomy education, medical students, medical training, anatomical education.
Nursing education	nursing education, nursing, nursing students, clinical skills, pressure injury, advanced cardiac life support, endotracheal aspiration.
Surgical and clinical training	surgery, surgical training, surgical education, neurosurgery, surgical simulator, clinical training, medical simulation, image-guided surgery.
Immersive representations and experiences	3d visualization, visualization, 3d models, 3d modeling, 3d computer graphics, 3d model, 3d technology, interactive computer graphics, visual representations, immersive technologies, immersive technology, immersive learning, immersive learning experience

## Data Availability

The original contributions presented in this study are included in the article; further inquiries can be directed to the first author.

## Public Involvement Statement

No public involvement in any aspect of this research.

## Guidelines and Standards Statement

The study followed the Preferred Reporting Items for Systematic reviews and Meta-Analyses (PRISMA 2020) reporting guidelines.
